# Biceps femoris accessory tendon tenodesis: A case report

**DOI:** 10.1002/ccr3.7984

**Published:** 2023-10-09

**Authors:** Scott Fong, Didi Wu, Lena Reed, Samantha Cheng, Kyle Cantave, Hanyu Chen, Patrick McGahan, James L. Chen

**Affiliations:** ^1^ Advanced Orthopedics and Sports Medicine San Francisco California USA; ^2^ Case Western Reserve University School of Medicine Cleveland OH USA

**Keywords:** biceps femoris, knee, orthopedics, snapping, sports medicine

## Abstract

**Key Clinical Message:**

We present a case of lateral knee pain from snapping of an accessory tendinous insertion of the biceps femoris. After failure of conservative treatment options, tenodesis of the accessory band to the direct arm insertion at the posterolateral edge of the fibular head effectively resolved symptoms.

**Abstract:**

There are several distinct causes of lateral knee pain including IT band syndrome, meniscus tears, or other soft tissue pathologies; however, a few case reports have shown the biceps femoris as a cause of lateral knee pain and snapping. Conservative treatment is of modest benefit to the patient in these scenarios, and an MRI is not always able to identify the accessory band, as in our case. Intraoperatively, we discovered an accessory band of the biceps femoris attaching to the anterolateral tibia, causing pain and snapping during knee flexion as the band passed over the fibular head. There have been various surgical attempts to address this pathology; however, we report a successful outcome after tenodesis of the accessory band to the direct insertion at the posterolateral fibular head.

## Graphical abstract

Tenodesis of the accessory band to the direct band of the biceps femoris tendon.

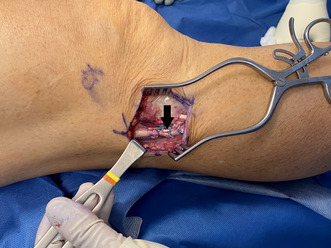

## 1 INTRODUCTION

Snapping at the lateral knee can be caused by a variety of pathologies involving surrounding soft tissue structures such as the popliteus, semitendinosus and/or gracilis tendons, IT Band syndrome, lateral meniscus tears, or rheumatoid nodules.^1^, ^2^, ^3^, ^4^ In rare instances, snapping lateral knee pain may also be caused by the distal tendon of the biceps femoris long head shifting over the fibular head.^5^, ^6^ Regardless, the various differential diagnoses should be properly ruled out by a careful history, a detailed physical exam, and advanced imaging.

Few cases of snapping biceps femoris tendons have been recorded in the literature across various case reports. Causes of the snapping biceps femoris tendon have been attributed to anomalous insertions (most prevalent),^7^, ^8^, ^9^, ^10^, ^11^, ^12^, ^13^, ^14^, ^15^, ^16^ tendon subluxation,^17^, ^18^ abnormalities of the fibular head,^19^, ^20^, ^21^, ^22^, ^23^ or secondary to trauma.^6^, ^24^ Conservative treatment is usually attempted first and consists of physical therapy and anti‐inflammatory medications. Surgery, which is usually the last resort and the most effective, consists of resecting the anomalous tendon insertion or correcting any fibular deformities.^7^, ^19^

In this report, we present a patient with lateral knee pain from an accessory insertion of the snapping biceps femoris tendon and discuss surgical exploration and repair. The patient was informed that his case would be submitted for publication and he provided consent.

## 2 CASE REPORT

**FIGURE 1:**
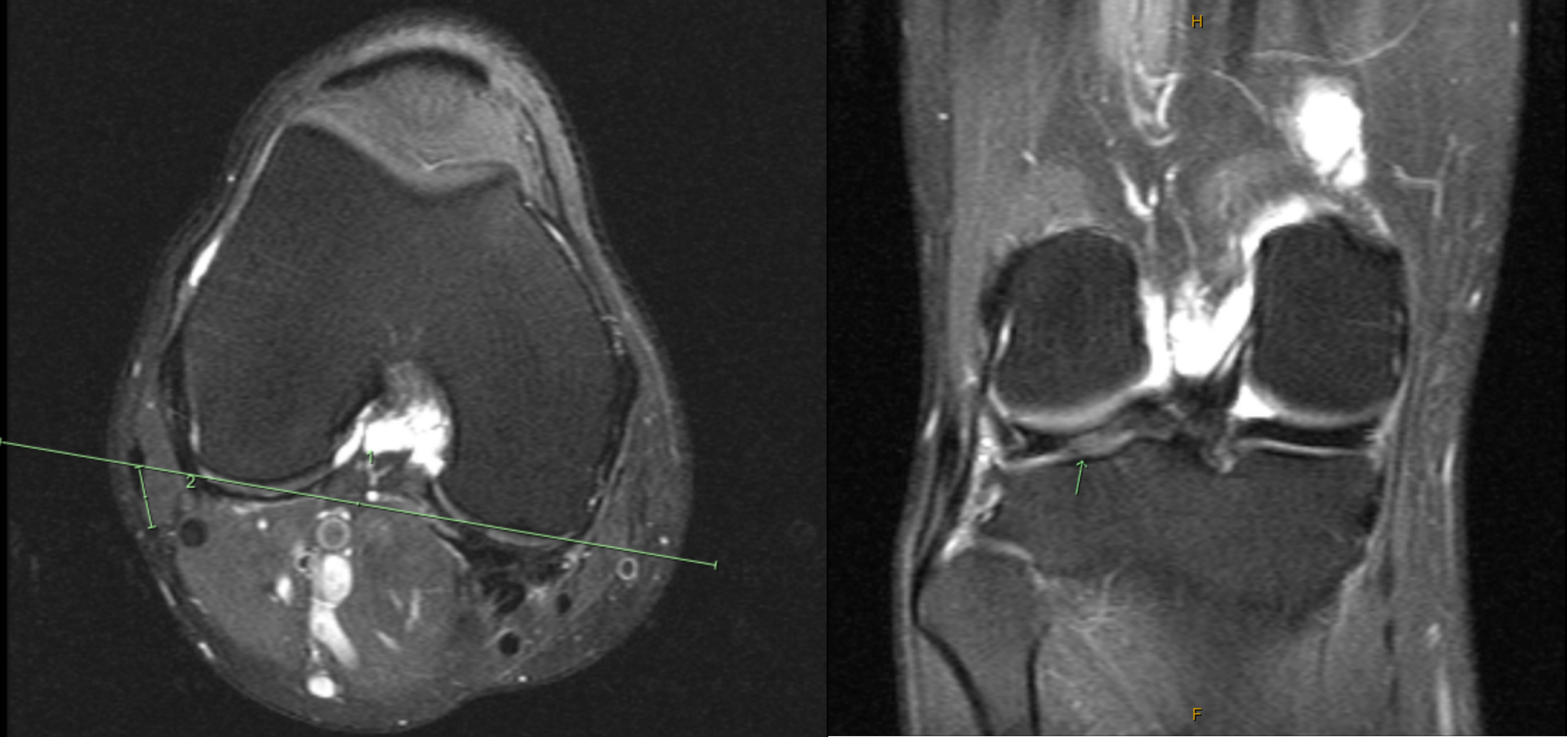
MRI of the right knee indicating no evidence of tear or peritendinous edema adjacent to the distal biceps femoris tendon or evidence of variant anatomy.

A 48 year‐old male patient presented to our clinic with lateral right knee snapping and pain that had been ongoing for over 2 years without trauma or other known cause. The patient noted the pain and snapping at the fibular head worsened with cycling and deep squats. On examination, no atrophy was noted; however, snapping was visible at knee flexion past 90 degrees. The patient had attempted activity modification, a home‐exercise program and icing without relief. Radiographs of the knee were unremarkable with no evidence of any osseous abnormalities or bony prominences at the fibular head. MRI imaging of the right knee did not indicate evidence of an anatomical variant or anomalous insertion of the biceps femoris tendon (Figure 1). After 8 weeks of physical therapy focused on knee conditioning and hamstring strengthening, the patient reported worsening of symptoms and elected to proceed with surgery as conservative options had been exhausted.

## 3 OPERATIVE FINDINGS AND TECHNIQUE

**FIGURE 2:**
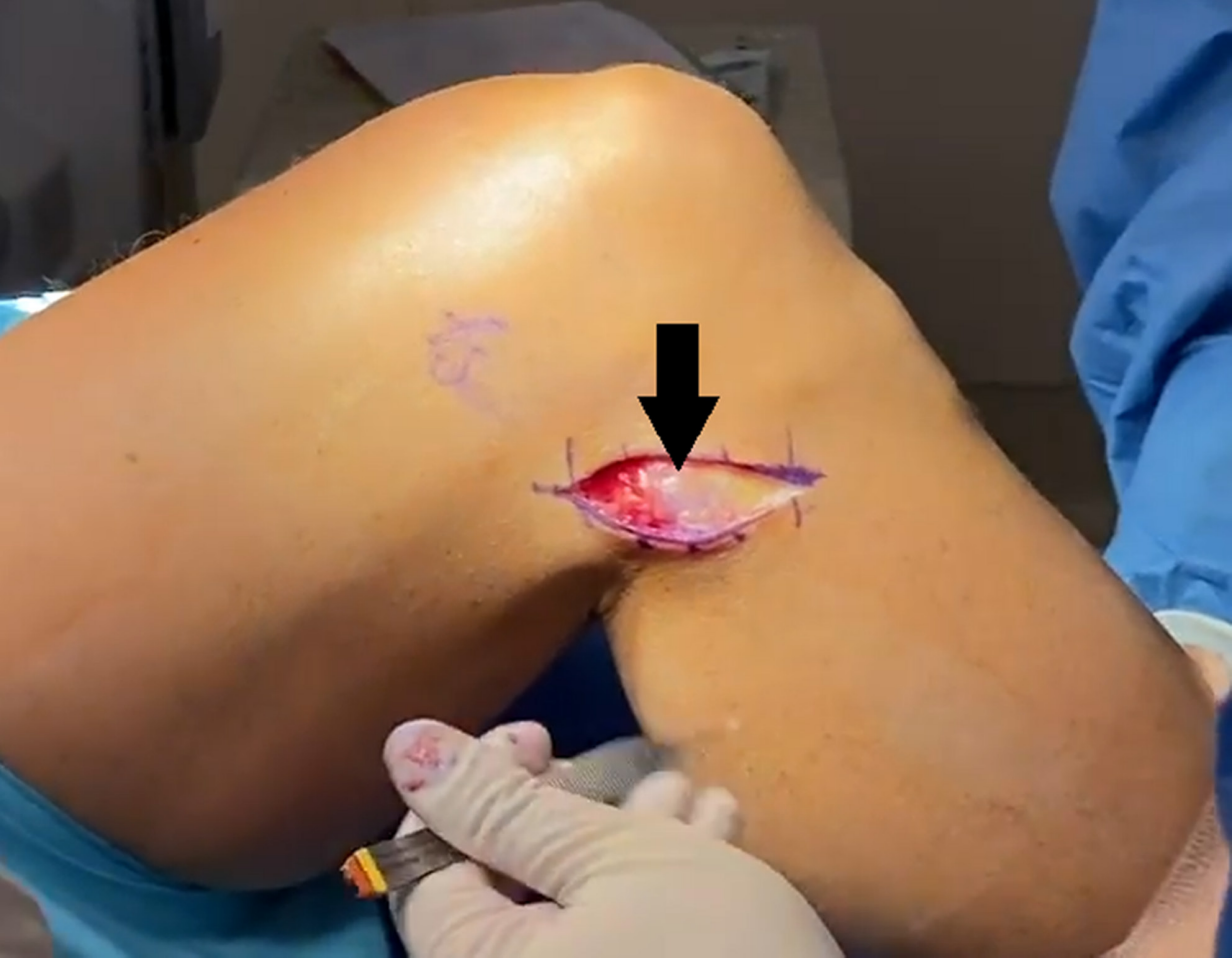
Intraoperative view of the lateral right knee flexed to 90 degrees with the patient positioned supine. The 4‐cm curvilinear incision is positioned squarely over the proximal fibula (arrow).

**FIGURE 3:**
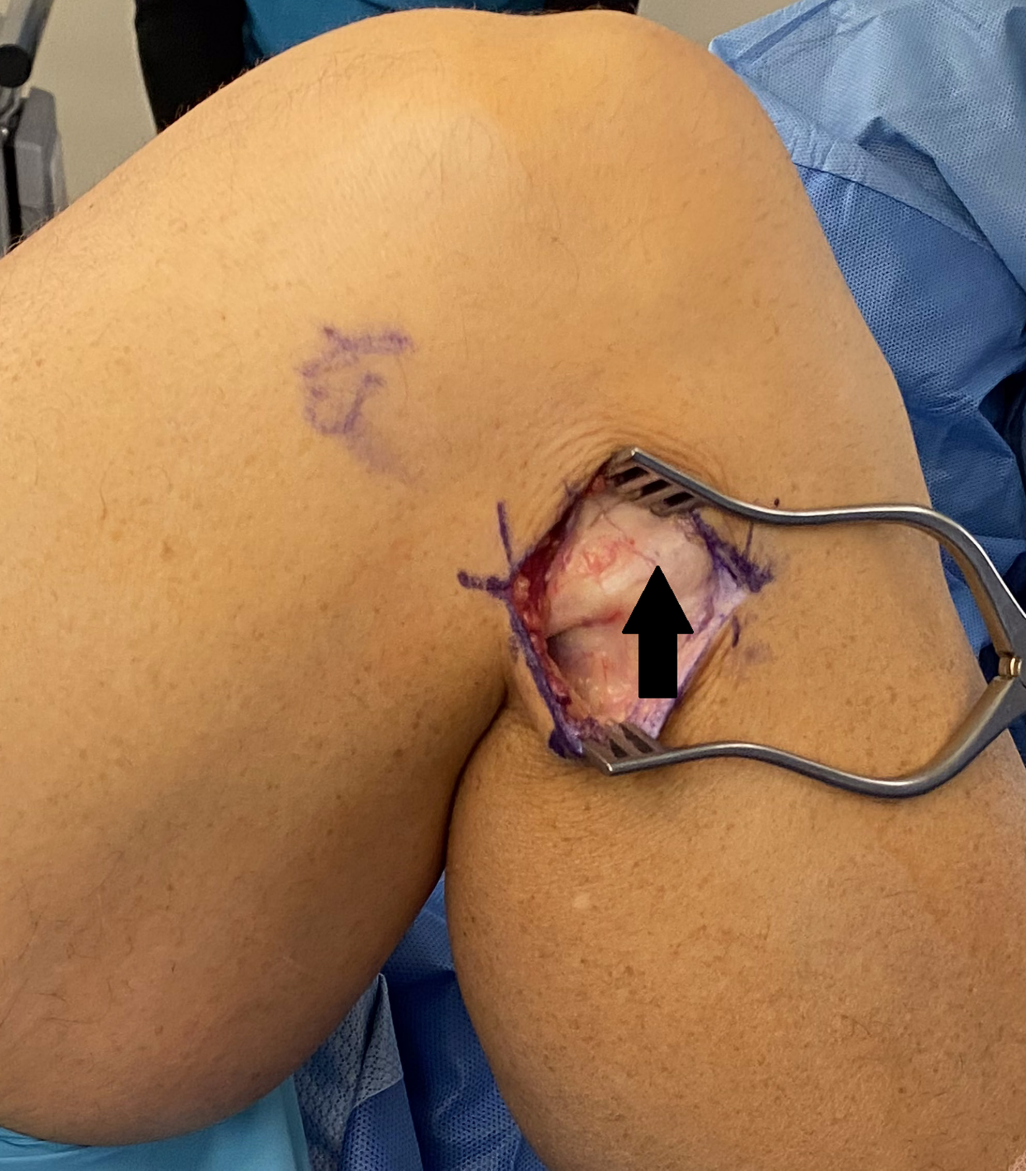
Flexed lateral knee. A thick band (arrow) can be visualized inserting on the anterolateral tibia.

**FIGURE 4:**
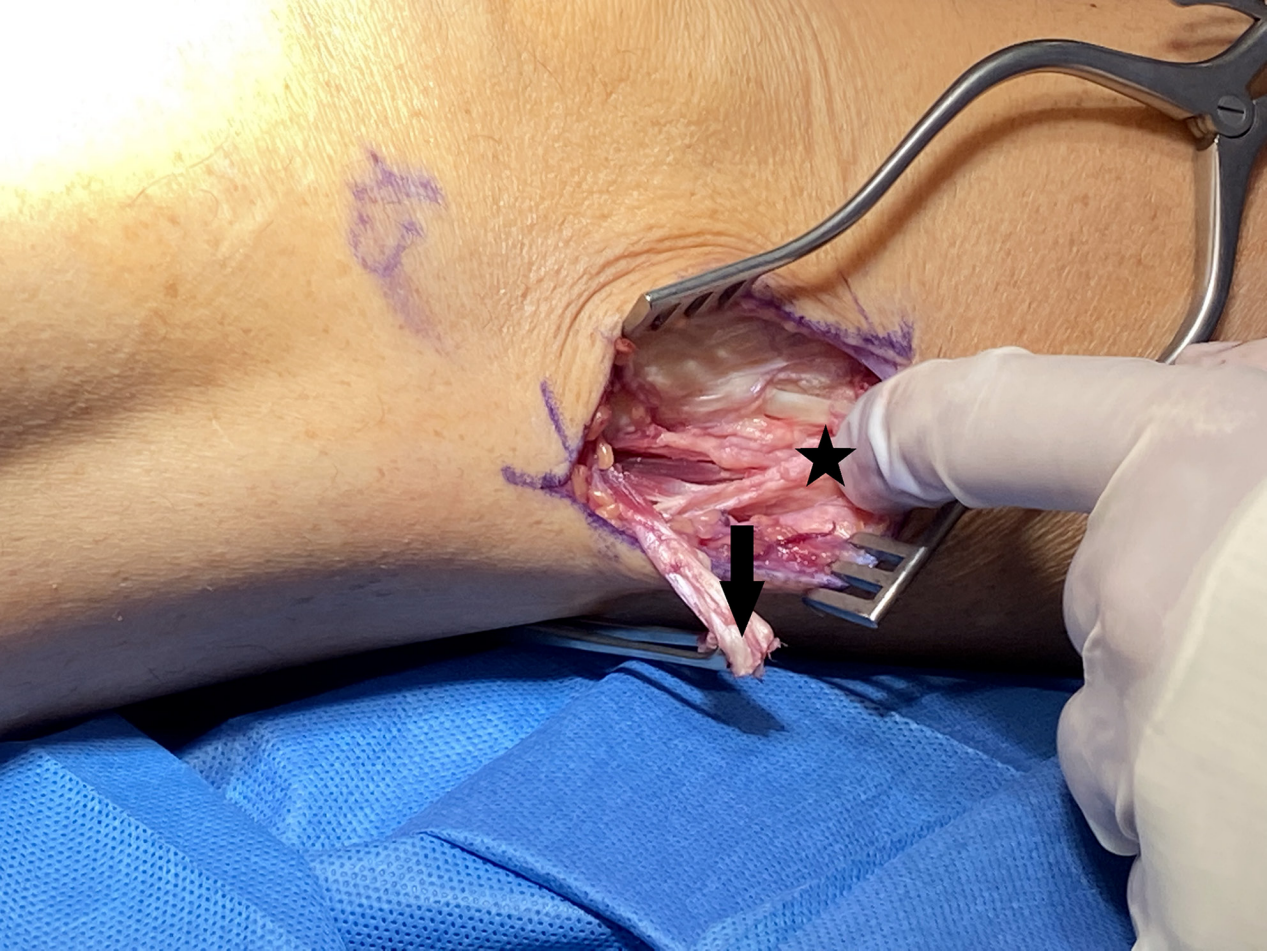
Right knee in full extension. The anomalous tendon insertion (arrow) has been resected and the thickness can be compared with that of the tendon's direct band (star).

**FIGURE 5:**
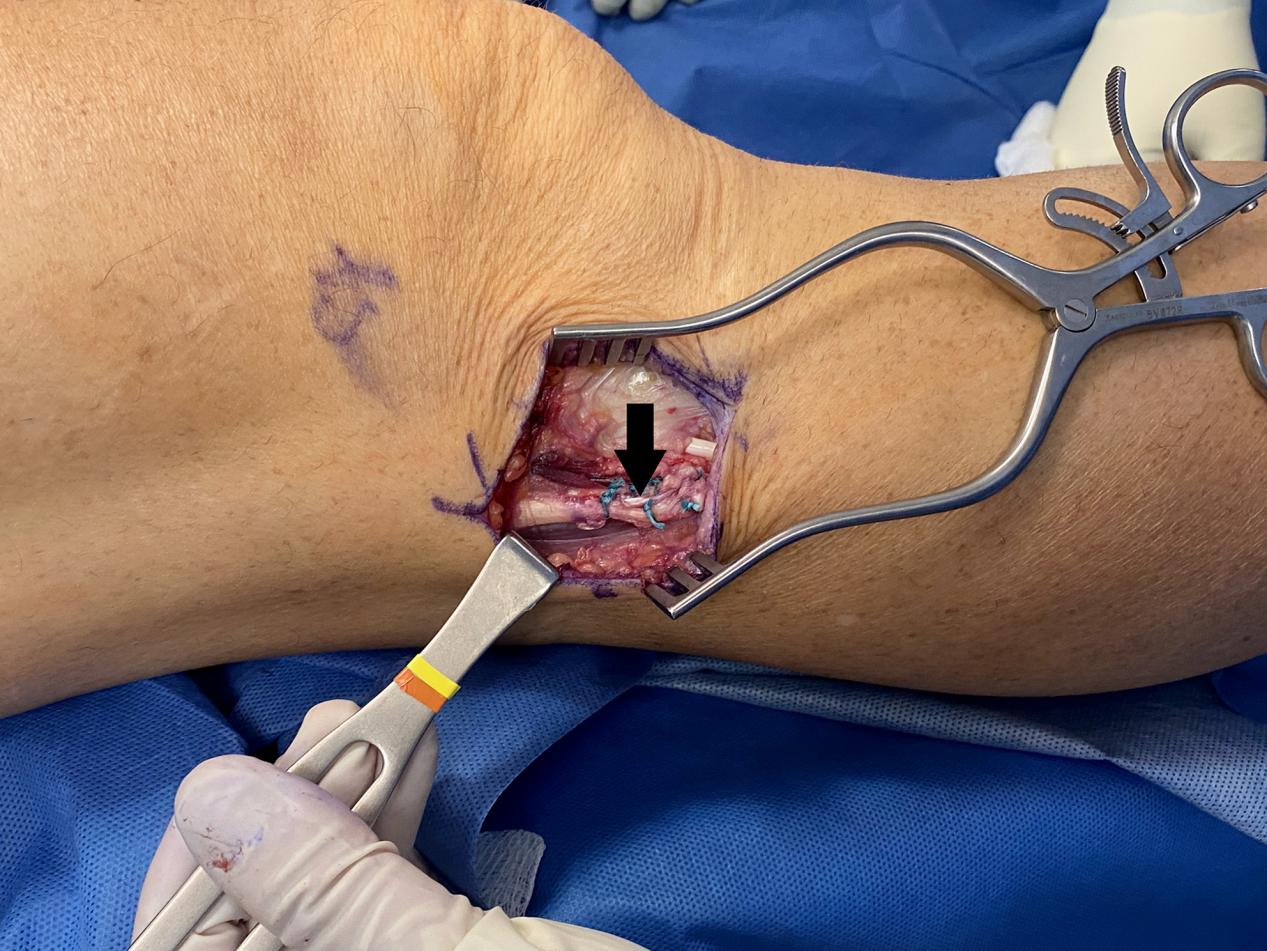
Right knee in full extension. The released anomalous insertion of biceps femoris tendon has been repaired down to the direct band of the tendon (arrow).

The patient was positioned supine on the operating table with a bump under the thigh. Preoperative antibiotics and general anesthesia with a peripheral nerve block were administered. After all bony landmarks were identified, a 4‐cm curvilinear incision was made over the proximal fibula (Figure 1). Careful dissection was taken down to the level of the biceps femoris tendon using Metzenbaum scissors and electrocautery. The self‐retaining retractors were used to better visualize the insertion of the tendon. Care was taken to protect the common peroneal nerve and other neurovascular structures. The tendon was inspected and a thick band was visualized inserting on the anterolateral tibia (Figure 2). The knee was then flexed to recreate the snapping and it was apparent that this band was the source of the snapping (Video S1). Upon further inspection, the direct band of the biceps femoris tendon was revealed. At this point, it was decided to dissect the anomalous insertion from the tibia (Figure 3) and repair this tendon down to the direct biceps femoris insertion on the posterior head of the fibula. A No. 2 Ethibond suture was used to secure the released tendon down on the direct band of the tendon (Figure 4). The released anomalous insertion of biceps femoris tendon was successfully repaired down to the direct band of the tendon (Figure 5). The knee was tested with flexion to ensure that there were no structures snapping at this time (Video S1).

At his first postoperative appointment, snapping at the lateral knee was visually confirmed to have been resolved. The patient was non‐weight bearing on the operative leg for one month after surgery and was counseled to regularly perform passive range of motion exercises to prevent stiffness. After 1 month, he transitioned to being full weight bearing and began physical therapy to regain full range of motion. At his 2‐month follow‐up, the patient's pain had completely resolved and he had full range of motion. He was able to resume his normal activities.

## 4 DISCUSSION

The anatomy of the biceps femoris tendon is complex and crucial to the biomechanical function of the knee. The muscle, composed of the short and long heads, is involved with hip extension, lateral rotation of the leg, and knee flexion.^25^ In addition, the biceps femoris plays an important role as a dynamic stabilizer of the knee and injury has been associated with rotatory instability of the knee.^25^ A cadaveric study of 56 knees by Salter et al. 2005 found that the biceps femoris tendon is composed of medial and lateral slips and was found to attach to the lateral condyle of the femur, popliteus, and the arcuate popliteal ligament.^26^ The long head of the biceps femoris originates at the ischial tuberosity and has two tendinous insertions. The first is a direct arm that attaches to the posterolateral fibular head and the second is an anterior arm that attaches to the lateral aspect of the fibular head or the lateral tibial plateau.^16^, ^26^ In our case, an anomalous attachment of the biceps femoris to the anterolateral tibia was repositioned to the direct arm insertion to resolve painful snapping of the tendon over the fibular head.

Due to the unremarkable findings on imaging, the diagnosis of a snapping knee due to an accessory tendon is difficult. There is some evidence to suggest that use of dynamic ultrasound may aid in making the diagnosis of a snapping biceps femoris tendon.^27^ However the diagnosis is often made clinically, as in our case, with positive findings presenting during the physical examination.^5^, ^10^

Various surgical approaches have been used to treat snapping of the biceps femoris tendon. One such approach is resection of the fibular head.^19^, ^20^ McNulty et al. successfully resolved symptoms by removing the prominent ridge on the posterior aspect of the fibular head, which caused snapping.^19^ Fung et al. reported a 17‐year‐old soccer player with bilateral exostoses at the fibular head treated surgically with exostosis excision, biceps tendon debridement, and fibular prominence smoothening with success.^20^

In other case reports, the anomalous tendon insertions may be resected.^5^, ^13^, ^16^ Fritsch et al.,^5^ reported an enlarged anterior arm of the biceps femoris tendon which elicited snapping. The thickened anterior arm was then detached and shuttled through a fibular tunnel, which resolved the snapping. Further, Reid et al.^16^ reported painful snapping in a 15‐year‐old athlete, which was resolved through resection of the accessory biceps femoris attachment and reinsertion into the fibular head with suture anchors and a Krackow suture. In Ernat et al.,^13^ the anterolateral tibial and thickened fibular accessory bands were released without reattachment, which resolved snapping at the lateral knee.

In Date et al.,^10^ an anomalous insertion of the biceps femoris at the anterolateral proximal tibia as well as the anterior arm at the lateral edge of the fibular head were sutured to the direct arm on the posterolateral fibular head using three stitches. Similar to Date's case, the accessory band of our patient's biceps femoris tendon was sutured against the direct arm and periosteum with only stitches without the use of suture anchors. In addition, given the crucial role of the biceps femoris to knee function, we felt that reattachment of the accessory biceps femoris tendon insertion was more appropriate than a tenotomy alone.

In our patient, conservative management with anti‐inflammatory medications and 2 months of physical therapy was initially attempted; however, these interventions failed to relieve his pain or snapping. Further, radiographs did not identify any abnormal features at or prominence of the fibular head that would have indicated a fibular head resection. Given the patient's visible, symptomatic lateral knee snapping and lack of relief from conservative treatment, the patient opted for surgical biceps femoris insertion exploration with possible accessory band release and transposition. Following surgery, our patient experienced successful resolution of symptoms and was able to return to an active lifestyle at 2 months follow‐up without recurrence of symptoms. Our unique case contributes to the existing literature by demonstrating an accessory anterolateral tibial insertion of the biceps femoris tendon as the underlying cause of painful snapping over the fibular head. Past cases of snapping biceps femoris tendons at the lateral knee have been treated uniquely depending on their pathophysiological root. When conservative treatments prove ineffective, surgical intervention emerges as a viable solution. In our case, symptoms were successfully resolved by the tenodesis of the accessory band to the direct arm insertion at the posterolateral edge of the fibular head.

## 5 CONCLUSION

Snapping of the biceps femoris tendon is a relatively rare occurrence that can cause painful and audible clicking that interferes with a patient's lifestyle. Conservative treatment is usually unable to resolve symptoms. There have been a variety of surgical approaches aimed at treating this anomaly. Accessory tendon tenodesis to the posterior head of the fibula may preserve knee stability and allow for quick recovery times and resolution of symptoms. We present a rare case of an anomalous insertion of the biceps femoris tendon that was resected and reattached surgically to resolve pain and snapping. Ultimately, further research is needed to evaluate the long‐term success of such surgeries and their effects on knee mobility.

## AUTHOR CONTRIBUTIONS

**Scott Fong:** Conceptualization; data curation; formal analysis; investigation; methodology; resources; visualization; writing – original draft. **Didi Wu:** Conceptualization; formal analysis; investigation; methodology; resources; visualization; writing – original draft. **Lena Reed:** Conceptualization; investigation; methodology; resources; visualization; writing – original draft. **Samantha L. Cheng:** Investigation; methodology; visualization; writing – review and editing. **Kyle Cantave:** Investigation; resources; visualization; writing – review and editing. **Hanyu Chen:** Investigation; writing – review and editing. **Patrick McGahan:** Conceptualization; investigation; writing – original draft; writing – review and editing. **James L. Chen:** Conceptualization; investigation; writing – original draft; writing – review and editing.

## FUNDING INFORMATION

None.

## CONFLICT OF INTEREST STATEMENT

J.L.C. is an educational consultant for Arthrex and receives compensation for lectures and instruction only.

## ETHICS STATEMENT

This study was performed in accordance with the ethical standards in the 1964 Declaration of Helsinki.

## CONSENT

The authors obtained written informed consent from the patient to publish this report in accordance with the patient consent policy established by *Clinical Case Reports*.

## Supporting information


Video S1

## Data Availability

All data generated or analyzed during this study are included in this published article and its supplementary information files.

## References

[1] Krause DA , Stuart MJ . Snapping popliteus tendon in a 21‐year‐old female. J Orthop Sports Phys Ther. 2008;38(4):191‐195. doi:10.2519/jospt.2008.2698 18434667

[2] Von Dercks N , Theopold JD , Marquass B , Josten C , Hepp P . Snapping knee syndrome caused by semitendinosus and semimembranosus tendons. A case report. Knee. 2016;23(6):1168‐1171. doi:10.1016/j.knee.2016.10.003 28340944

[3] Barker JU , Strauss EJ , Lodha S , Bach BR Jr . Extra‐articular mimickers of lateral meniscal tears. Sports Health. 2011;3(1):82‐88. doi:10.1177/1941738110385997 23015995 PMC3445190

[4] Chu A , Ginat D , Terzakis J , Seneviratne A , Schneider KS . Chronic sarcoid arthritis presenting as an intra‐articular knee mass. J Clin Rheumatol. 2009;15(4):190‐192. doi:10.1097/RHU.0b013e3181a61c29 19455060

[5] Fritsch BA , Mhaskar V . Anomalous biceps femoris tendon insertion leading to a snapping knee in a young male. Knee Surg Relat Res. 2017;29(2):144‐149. doi:10.5792/ksrr.15.067 28545180 PMC5450577

[6] Bansal R , Taylor C , Pimpalnerkar AL . Snapping knee: an unusual biceps femoris tendon injury. Knee. 2005;12(6):458‐460. doi:10.1016/j.knee.2004.12.010 16006128

[7] Lokiec F , Velkes S , Schindler A , Pritsch M . The snapping biceps femoris syndrome. Clin Orthop Relat Res. 1992;283:205‐206. 1395247

[8] Hernandez JA , Rius M , Noonan KJ . Snapping knee from anomalous biceps femoris tendon insertion: a case report. Iowa Orthop J. 1996;16:161‐163. 9129290 PMC2378142

[9] Kristensen G , Nielsen K , Blyme PJ . Snapping knee from biceps femoris tendon. A case report. Acta Orthop Scand. 1989;60(5):621. doi:10.3109/17453678909150135 2603666

[10] Date H , Hayakawa K , Nakagawa K , Yamada H . Snapping knee due to the biceps femoris tendon treated with repositioning of the anomalous tibial insertion. Knee Surg Sports Traumatol Arthrosc. 2012;20(8):1581‐1583. doi:10.1007/s00167-011-1778-4 22109681 PMC3402668

[11] Matar HE , Farrar NG . Snapping biceps femoris: clinical demonstration and operative technique. Ann R Coll Surg Engl. 2018;100(3):e59‐e61. doi:10.1308/rcsann.2018.0007 29364023 PMC5930108

[12] Bagchi K , Grelsamer RP . Partial fibular head resection for bilateral snapping biceps femoris tendon. Orthopedics. 2003;26(11):1147‐1149. doi:10.3928/0147-7447-20031101-17 14627114

[13] Ernat JJ , Galvin JW . Snapping biceps femoris tendon. Am J Orthop. 2018;47(7). doi:10.12788/ajo.2018.0055 30075041

[14] Bernhardson AS , LaPrade RF . Snapping biceps femoris tendon treated with an anatomic repair. Knee Surg Sports Traumatol Arthrosc. 2010;18(8):1110‐1112. doi:10.1007/s00167-009-1018-3 20020099

[15] Kissenberth MJ , Wilckens JH . The snapping biceps femoris tendon. Am J Knee Surg. 2000;13(1):25‐28. 11826921

[16] Reid L , Mofidi A . Bilateral snapping biceps femoris tendon: a case report and review of the literature. Eur J Orthop Surg Traumatol. 2019;29(5):1081‐1087. doi:10.1007/s00590-019-02392-9 30770981

[17] Crow SA , Quach T , McAllister DR . Partial tendon release for treatment of a symptomatic snapping biceps femoris tendon: a case report. Sports Health. 2009;1(5):435‐437. doi:10.1177/1941738109338360 23015904 PMC3445169

[18] Vavalle G , Capozzi M . Symptomatic snapping knee from biceps femoris tendon subluxation: an unusual case of lateral pain in a marathon runner. J Orthop Traumatol. 2010;11(4):263‐266. doi:10.1007/s10195-010-0117-8 21127937 PMC3014471

[19] McNulty M , Carreau J , Hendrickson N , Bollier M . Case report: snapping biceps femoris tendon due to abnormal fibular morphology. Iowa Orthop J. 2017;37:81‐84. 28852339 PMC5508271

[20] Fung DA , Frey S , Markbreiter L . Bilateral symptomatic snapping biceps femoris tendon due to fibular exostosis. J Knee Surg. 2008;21(1):55‐57. doi:10.1055/s-0030-1247793 18300673

[21] Bach BR Jr , Minihane K . Subluxating biceps femoris tendon: an unusual case of lateral knee pain in a soccer athlete. A case report. Am J Sports Med. 2001;29(1):93‐95. doi:10.1177/03635465010290012101 11206264

[22] Hadeed MM , Post M , Werner BC . Partial Fibular Head Resection Technique for Snapping Biceps Femoris. Arthrosc Tech. 2018;7(8):e859‐e862. doi:10.1016/j.eats.2018.04.008 30167365 PMC6112230

[23] Zadeh C , Khoury N , Najem E , Moukaddam H . Snapping of bilateral biceps femoris tendons: a case report and brief review. Radiol Case Rep. 2022;17(4):1293‐1299. doi:10.1016/j.radcr.2021.12.058 35242255 PMC8857580

[24] Saltzman BM , Collins MJ , Arns TA , Forsythe B . Unilateral snapping biceps femoris tendon with an anomalous insertion treated with anatomic repositioning and lengthening with a single suture anchor: a report of two cases. JBJS Case Connect. 2018;8(1):e13. doi:10.2106/JBJS.CC.16.00251 29489524

[25] Terry GC , LaPrade RF . The biceps femoris muscle complex at the knee: its anatomy and injury patterns associated with acute anterolateral‐anteromedial rotatory instability. Am J Sports Med. 1996;24(1):2‐8. 8638749 10.1177/036354659602400102

[26] Tubbs RS , Caycedo FJ , Oakes WJ , Salter EG . Descriptive anatomy of the insertion of the biceps femoris muscle. Clin Anat. 2006;19(6):517‐521. doi:10.1002/ca.20168 16283645

[27] Guillin R , Mendoza‐Ruiz JJ , Moser T , Ropars M , Duvauferrier R , Cardinal E . Snapping biceps femoris tendon: a dynamic real‐time sonographic evaluation. J Clin Ultrasound. 2010 Oct;38(8):435‐437. doi:10.1002/jcu.20728 20658565

