# Supplementary material for: Biceps femoris accessory tendon tenodesis: A case report
Source: Clin Case Rep. 2023 Oct 9;11(10):e7984. doi: 10.1002/ccr3.7984 (PMC10562657; doi:10.1002/ccr3.7984)
Supplement: Supplementary file 1 — Video S1 [file CCR3-11-e7984-s001.zip › ccr37984-sup-0002-Legend.docx]

Video Legend: [Video 1]

- 0:08 Preoperative snapping biceps femoris tendon over fibular head range of motion with squat.
- 0:16 Preoperative snapping biceps femoris over fibular head range of motion under anesthesia.
- 0:22 Tendon dissection and release.
- 0:58 Tenodesis to inferior tendon band.
- 1:17 Snug repair of excess/loose tendon.
- 1:30 Final repair.
- 1:41 Postoperative range of motion under anesthesia.
- 1:46 Postoperative range of motion with squat.
